# Economic burden of illness of acute coronary syndromes: medical and productivity costs

**DOI:** 10.1186/1472-6963-11-35

**Published:** 2011-02-14

**Authors:** Zhenxiang Zhao, Melissa Winget

**Affiliations:** 1 Global Health Outcomes, Eli Lilly and Company, 1400 West Raymond Street, Indianapolis, IN 46221, USA; 2 Market Access and Reimbursement (MAR), i3 Innovus, PO Box 9472, Minneapolis, MN 55440-9472, USA

## Abstract

**Background:**

The significant economic burden associated with acute coronary syndromes (ACS) provides a need to evaluate both medical costs and productivity costs, according to evolving guideline-driven ACS treatment strategies, medical management (MM), percutaneous coronary intervention (PCI), or coronary artery bypass graft (CABG).

**Methods:**

Commercially insured individuals, aged 18-64, with an emergency room (ER) visit or hospitalization accompanied by an ACS diagnosis (index event) were identified from a large claims database between 01/2004 and 12/2005 with a 1-year follow-up period. Patients who had an ACS diagnosis in the 12 months prior to their index event were excluded. Patients were divided into 3 groups according to treatment strategies during the index event: MM, PCI, or CABG. A subset of patients was identified for the productivity cost analysis exploring short-term disability and absenteeism costs. Multivariate generalized linear models were performed to examine the ACS costs by 3 different treatment strategies.

**Results:**

A total of 10,487 patients were identified for the medical cost analysis. The total 1-year medical costs (index event costs plus the 1-year follow-up costs) were lowest for MM patients ($34,087), followed by PCI patients ($52,673) and CABG patients ($86,914). Of the 3,080 patients in the productivity costs analysis, 2,454 patients were identified in the short-term disability cohort and 626 patients were identified in the absenteeism cohort. Both the estimated mean total 1-year short-term disability and absenteeism costs were highest for CABG patients ($17,335, $14,960, respectively) compared to MM patients ($6,048, $9,826, respectively) and PCI patients ($9,221, $9,460, respectively).

**Conclusions:**

Both total 1-year medical costs and 1-year productivity costs are substantial for working-aged individuals with ACS. These costs differ according to the type of treatment strategy, with CABG having higher costs compared to either PCI or MM.

## Background

Acute coronary syndromes (ACS), including unstable angina (UA), non-ST-segment elevation myocardial infarction (NSTEMI), and ST-segment elevation myocardial infarction (STEMI), are common and costly. In 2006 alone, it was estimated that 733,000 hospital discharges were due to ACS, increasing to 1,365,000 when adding secondary discharges [1]. The morbidity and mortality associated with ACS are substantial, as half of all deaths due to cardiovascular disease are attributed to ACS [2]. Further, the economic impact of ACS has been estimated as high as $150 billion annually [2]. With the large number of patients impacted by ACS, it becomes increasingly important to examine the economic burden of this illness in terms of both medical and productivity costs.

The substantial costs associated with ACS include treatment related medical costs, as well as costs due to loss of productivity [3]. Previous studies evaluating medical costs have shown total first-year treatment cost estimates to be $22,528 to $32,345, with the majority of these costs due to hospitalizations [4-6]. The American Heart Association estimates that loss of productivity for coronary heart disease will account for nearly $81.1 billion in 2009, and there are limited data available on the productivity costs of ACS specifically [1]. With prior studies concentrating on the medical costs associated with ACS, there is an unmet need for research exploring the impact of productivity costs.

Analyzing ACS costs by different treatment strategies should be of special interest to healthcare professionals as different treatment strategies are important drivers of ACS costs and may have different implications on healthcare resource utilization and productivity loss of patients. Depending on the different manifestations of ACS, UA/NSTEMI versus STEMI, the current 2007 American College of Cardiology/American Heart Association (ACC/AHA) guidelines allow conservative noninterventional medical management (MM) and/or invasive interventional management by percutaneous coronary intervention (PCI) or coronary artery bypass graft (CABG) [7,8]. There are limited data available on the effects of different treatment strategies on the costs of ACS.

Over the years, the ACC/AHA guidelines have been updated to reflect new data and clinical trial results. Previous studies investigating the cost of illness for ACS, however, have focused on data from the late 1990s or early 2000s, prior to the results of the Clopidogrel in Unstable angina to prevent Recurrent Events (CURE) trial for clopidogrel [9], as well as several versions of the ACC/AHA guidelines. With the implementation of the updated ACC/AHA guidelines and the increased use of clopidogrel, the outcomes and costs associated with ACS may have changed. The goals of this study were to estimate both 1-year medical costs and productivity (short-term disability and absenteeism) costs by using more recent data, and to assess the effects of treatment strategies (MM, PCI, and CABG) during the index ACS event on costs.

## Methods

### Data Source

These retrospective claims database analyses were performed using two of the Thomson Reuters MarketScan^® ^Research Databases. For the medical cost analyses, the MarketScan Commercial Claims and Encounters (CCAE) database was used. The CCAE database captures individual-level clinical utilization, costs, and enrollment across inpatient, outpatient and prescription drug categories from a selection of large United States (US) employers with contributing health plans. The MarketScan Health and Productivity Management (HPM) database was used for the productivity cost analyses. The HPM database is a subset of the CCAE database and contains information on short-term disability, absence, and workers' compensation experience that is linkable to the medical, pharmacy, and enrollment data in MarketScan databases.

### Study Design

Medical claims data were extracted from January 2003 to December 2006 for individuals, aged 18-64, that were seen in an emergency room (ER) or hospitalized between January 2004 and December 2005 with a diagnosis of ACS, based on ICD-9-CM codes 410.xx and 411.1. The first ACS-related ER visit or hospitalization was considered as the "index event." Patients who had an ACS diagnosis one year prior to the index event were excluded from the study. Patients were required to have a minimum 12 months of continuous insurance eligibility prior to and post the index event. Patients were categorized into three groups according to their ACS treatment strategy during the index event: MM, PCI, or CABG. For the subset productivity cost analyses, patients were required to have had a minimum of 12-months eligibility for incurring short-term disability and absence in the HPM database. Data for these individuals were linked to their employers' short-term disability and absenteeism records via unique encrypted personal identification numbers provided by the employers.

### Data Analyses

All statistical analyses were performed with Stata 9 software (College Station, Texas, USA) and an *a priori *p-value of 0.05 was considered statistically significant. Descriptive analyses according to MM, PCI, and CABG were performed on patient demographic and clinical characteristics using chi-square tests for categorical variables and *t *tests for continuous variables. The demographic characteristics included age, gender, insurance plan type (see Appendix for description), and geographic region. The clinical characteristics included comorbidities, the Charlson comorbidity score [10], primary admission diagnosis, length of stay, and discharge mortality rate.

Medical costs were categorized into costs incurred during the index event and costs incurred during the 1-year follow-up period for MM, PCI, and CABG patients. For the 1-year follow-up costs, costs were further divided into inpatient, outpatient, and pharmacy costs. All costs were reported in 2005 US dollars. For the productivity cost analyses, short-term disability and absenteeism costs incurred during the 1-year follow-up period were estimated for the MM, PCI, and CABG groups. These productivity costs were further divided into two categories for each group: the costs incurred within the first 30 days of the index event and the costs incurred from day 31 through one year. The hourly value of $42.67 of productivity losses was estimated based on the average hourly wage and benefits rate reported in the 1999 Medstat Benchmark Survey of Hourly Compensation and inflated using the 2005 Bureau of Labor Statistics Employment Cost Index for Private Industry [11]. Differences in unadjusted medical and productivity costs were descriptively compared across MM, PCI, and CABG groups by using nonparametric Wilcoxon rank sum tests. To account for the non-negativity and skewed distribution of costs and short-term disability and absenteeism days and to avoid heteroscedasticity in simple least-squares models, generalized linear models (GLM) with log link function and gamma distribution were used [12-15]. Specifically, multivariate GLM were performed to study the effects of treatment strategies on the total 1-year medical costs and productivity costs, while controlling for patient characteristics including age, gender, insurance plan type, Charlson comorbidity score, primary admission diagnosis, and treatment strategy. The cost ratio (the exponential of the beta coefficient from the GLM estimation) was presented to show the relative increase in mean costs by increasing covariates by 1 unit [15]. Statistical bootstrapping was used to obtain 95% confidence intervals.

## Results

### Patient Characteristics

A total of 10,487 ACS patients was identified and divided into three mutually exclusive groups according to treatment strategy during their index event: MM (n = 4,894), PCI (n = 4,729), and CABG (n = 864). Patient demographic and clinical characteristics for the total population are displayed in Table 1. The average ages for MM, PCI, and CABG patients were 54.3, 54.8, and 56.5, respectively. Overall, patients in the MM group were significantly (p < 0.05) more likely to be female, be younger, and have a higher mean Charlson comorbidity score compared to the PCI and CABG groups. In addition, patients in the PCI and CABG groups had significantly (p < 0.05) longer mean lengths of stay compared to MM patients during the index event: 3.14 days, 8.65 days, and 1.63 days, respectively.

**Table 1:** Patient demographic and clinical characteristics by ACS index event treatment strategy

Variables	MM n = 4,894	PCI n = 4,729	CABG n = 864	p-value*
Age, mean (SD)	54.3 (7.3)	54.8 (6.6)	56.5 (5.7)	a,b,c

Age group (%)				

18-34 years	1.6	0.7	0.1	a,b,c
	
35-44 years	9.1	7.1	3.8	
	
45-54 years	32.3	34.0	28.0	
	
55-64 years	57.0	58.2	68.1	

Gender (%)				

Female	42.2	21.0	22.1	b,c
	
Plan Type (%)				

Comprehensive	19.1	17.0	19.4	b,c
	
HMO	21.0	16.0	14.0	
	
PPO	46.9	52.6	52.7	
	
POS	12.5	13.7	13.2	
	
Unknown	0.5	0.7	0.7	

Region (%)				

Northeast	8.2	7.0	5.7	n.s.
	
Northcentral	35.6	36.2	35.9	
	
South	40.0	41.2	44.7	
	
West	15.9	15.2	13.4	
	
Unknown	0.4	0.3	0.3	

Comorbidities (%)				

Diabetes without chronic complications	25.0	18.2	24.9	a,b

Diabetes with chronic complications	3.1	1.4	2.1	b

Congestive heart failure	7.5	2.0	2.4	b,c

Cerebrovascular disease	7.0	3.2	4.4	b,c

Peripheral vascular disease	3.4	1.8	1.9	b,c

Renal disease	3.4	1.3	1.6	b,c

Rheumatologic disease	2.1	1.3	1.7	b

Peptic ulcer disease	0.8	0.5	0.5	n.s.

Chronic pulmonary disease	14.2	10.2	8.8	b,c

Any malignancy	4.4	3.4	3.7	b

Metastatic solid tumor	0.6	0.3	0.1	b

Mild liver disease	0.6	0.2	0.2	b

Moderate or severe liver disease	0.2	0.06	0.1	b

AIDS	0.2	0.2	0	n.s.

Dementia	0.2	0.1	0.1	n.s.

Hemiplegia or paraplegia	0.2	0.02	0.1	b

Mean (SD) Charlson Comorbidity Score	0.75 (1.16)	0.48 (0.89)	0.56 (0.88)	a,b,c

MI listed as the primary diagnosis (%)	34.2	69.9	57.9	a,b,c

Length of stay (days)				

Mean (SD)	1.63 (3.01)	3.14 (2.41)	8.65 (5.15)	a,b,c

Median	1	3	8	

75th percentile	2	4	10	

Mortality rate at hospital discharge	0.2	0.1	0.2	n.s.

*n.s. = not significant; a = p < 0.05 CABG vs. PCI; b = p < 0.05 MM vs. PCI; c = p < 0.05 MM vs. CABG.

CABG = coronary artery bypass graft; HMO = health maintenance organization; MI = myocardial infarction; MM = medical management; PCI = percutaneous coronary intervention; POS = point of service; PPO = preferred provider organization; SD = standard deviation.

For the productivity cost analysis, 3,080 patients had available data for analysis with 2,454 patients in the short-term disability cohort (MM: n = 1,169, PCI: n = 1,034, and CABG: n = 251) and 626 patients in the absenteeism cohort (MM: n = 337, PCI: n = 230, and CABG: n = 59). In both cohorts, MM patients were more likely to be female, be younger, and have a higher mean Charlson comorbidity score compared to PCI and CABG patients.

### Medical Costs

Table 2 presents the total medical costs (index event costs plus 1-year follow-up costs) for the MM, PCI, and CABG groups. Index event costs were lowest for MM patients ($8,905), followed by PCI patients ($31,379) and CABG patients ($63,909), respectively (p < 0.05). Index event costs for the interventional treatments accounted for 59.6% (PCI) and 73.5% (CABG) of the total medical costs for those groups, and 26.1% of the total medical costs for the MM group. Of the total 1-year follow-up costs, rehospitalization costs accounted for 45.3% for the MM group, 41.2% for the PCI group, and 43.5% for the CABG group, with ACS-related rehospitalization costs accounting for 18.5%, 17.4%, and 15.2%, respectively. ACS-related prescription drug costs accounted for 4.9%, 10.9% and 6.6% of the 1-year follow-up costs for MM, PCI, and CABG patients, respectively. The total costs obtained by adding the index event costs to the 1-year follow-up costs were lowest for MM patients ($34,087), followed by PCI patients ($52,673) and CABG patients ($86,914), respectively (p < 0.05).

**Table 2:** Mean total medical costs by ACS index event treatment strategy

Variables	MM n = 4,894	PCI n = 4,729	CABG n = 864	p-value*
**Index Event Costs, $ (SD)**	8,905 (14,832)	31,379 (22,569)	63,909 (43,620)	a,b,c

**1-Year Follow-Up Costs, $ (SD)**				

Inpatient	11,397 (35,335)	8,781 (23,877)	10,017 (35,177)	b,c

Outpatient	10,583 (20,956)	8,883 (20,693)	9,980 (21,077)	a,b

Pharmacy	3,202 (4,031)	3,630 (3,128)	3,008 (3,058)	a,b,c

Total 1-Year Follow-Up Costs	25,182 (45,625)	21,294 (35,327)	23,005 (46,999)	n.s.

**Total Medical Costs, $ (SD)**	34,087 (49,958)	52,673 (44,957)	86,914 (69,836)	a,b,c

*n.s. = not significant; a = p < 0.05 CABG vs. PCI; b = p < 0.05 MM vs. PCI; c = p < 0.05 MM vs. CABG.

CABG = coronary artery bypass graft; MM = medical management; PCI = percutaneous coronary intervention; SD = standard deviation.

Table 3 displays the results from the multivariate regression analysis for the mean total 1-year medical costs. Female gender was significantly (p < 0.05) associated with lower 1-year costs. All other parameters were significantly (p < 0.05) associated with higher 1-year costs: increasing age, insurance plan type other than a health maintenance organization (HMO), higher Charlson comorbidity scores, myocardial infarction (MI) as the primary admission diagnosis, and interventional treatment strategies (PCI and CABG). Controlling for patient characteristics, the estimated mean total 1-year medical costs were calculated to be $39,017 (95% CI: $36,332-$41,702) for MM patients, $58,313 (95% CI: $54,972-$61,653) for PCI patients, and $91,977 (95% CI: $85,044-$98,910) for CABG patients.

**Table 3:** Results from multivariate regression for mean total 1-year medical costs

Variables	Cost Ratio	95% CI	p-value
Age Groups			

Age 45-54 (vs. ≤44 years)	1.109	1.029 - 1.195	0.006

Age 55+ (vs. ≤44 years)	1.187	1.109 - 1.269	< 0.0001

Gender			

Female	0.906	0.869 - 0.945	< 0.0001

Plan Type			

Comprehensive (vs. HMO)	1.084	1.018 - 1.155	0.01

PPO (vs. HMO)	1.246	1.179 - 1.318	< 0.0001

POS (vs. HMO)	1.116	1.040 - 1.198	0.002

Charlson score = 1 (vs. Charlson score = 0)	1.133	1.092 - 1.176	< 0.0001

Charlson score > 1 (vs. Charlson score = 0)	1.311	1.237 - 1.389	< 0.0001

MI listed as the primary diagnosis	1.351	1.302 - 1.402	< 0.0001

CABG (vs. MM)	2.693	2.519 - 2.879	< 0.0001

PCI (vs. MM)	1.595	1.521 - 1.674	< 0.0001

Prior 1-year health care costs	1.000	1.000 - 1.000	< 0.0001

CABG = coronary artery bypass graft; CI = confidence interval; HMO = health maintenance organization; MI = myocardial infarction; MM = medical management; PCI = percutaneous coronary intervention; POS = point of service; PPO = preferred provider organization.

### Productivity Costs

For the 1-year productivity costs, Table 4 shows that patients in the CABG group had significantly (p < 0.05) higher 1-year short-term disability costs ($17,335) and absenteeism costs ($14,960) compared to MM patients (short-term disability costs: $6,048 and absenteeism costs: $9,826) and PCI patients (short-term disability costs: $9,221 and absenteeism costs: $9,460). The majority of the short-term disability costs occurred within the first 30 days of the index event for all three groups. In contrast, the majority of the absenteeism costs for all three groups occurred after 30 days from the index event, between day 31 and the end of the 1-year follow-up period.

**Table 4:** Mean 1-year productivity costs by ACS index event treatment strategy

Variables	MM	PCI	CABG	p-value*
**Short-Term Disability Costs, $ (SD)**	**n = 1,169**	**n = 1,034**	**n = 251**	

≤30 days from index hospitalization	3,578 (10,131)	6,923 (13,510)	15,995 (19,100)	a,b,c

> 30 days - 1 year from index hospitalization	2,470 (8,455)	2,298 (8,006)	1,340 (6,339)	a,c

Total Short-Term Disability Costs	6,048 (13,446)	9,221 (15,631)	17,335 (19,987)	a,b,c

**Absenteeism Costs, $ (SD)**	**n = 337**	**n = 230**	**n = 59**	

≤30 days from index hospitalization	1,505 (2,038)	1,767 (2,257)	2,627 (2,995)	n.s.

> 30 days - 1 year from index hospitalization	8,320 (8,379)	7,692 (7,581)	12,332 (12,281)	a,c

Total Absenteeism Costs	9,826 (9,808)	9,460 (9,067)	14,960 (14,623)	a,c

*n.s. = not significant; a = p < 0.05 CABG vs. PCI; b = p < 0.05 MM vs. PCI; c = p < 0.05 MM vs. CABG.

CABG = coronary artery bypass graft; MM = medical management; PCI = percutaneous coronary intervention; SD = standard deviation.

Tables 5 and 6 present the results of the multivariate regression analysis for the mean total 1-year short-term disability costs and mean total 1-year absenteeism costs, respectively. For the short-term disability cohort, female gender was significantly (p < 0.05) associated with lower costs and MI as the primary admission diagnosis and interventional treatment strategies (PCI and CABG) were significantly (p < 0.05) associated with higher costs. For the absenteeism cohort, female gender and insurance plan type other than an HMO were significantly (p < 0.05) associated with lower costs, while age (45-54 vs. ≤44 years) and CABG (vs. MM) were both significantly (p < 0.05) associated with higher costs. Controlling for patient characteristics, the estimated mean total 1-year short-term disability costs were $5,937 (95% CI: $4,293-$7,582) for MM patients, $9,400 (95% CI: $7,084-$11,718) for PCI patients, and $18,051 (95% CI: $13,435-$22,668) for CABG patients. The estimated mean total 1-year absenteeism costs were $9,733 (95% CI: $7,501-$11,966) for MM patients, $9,785 (95% CI: $7,731-$12,138) for PCI patients, and $13,958 (95% CI: $9,649-$18,266) for CABG patients.

**Table 5:** Results from multivariate regression for mean total 1-year short-term disability costs

Variables	Cost Ratio	95% CI	p-value
Age Groups			

Age 45-54 (vs. ≤44 years)	1.065	0.816 - 1.389	0.645

Age 55+ (vs. ≤44 years)	0.991	0.771 - 1.274	0.942

Gender			

Female	0.675	0.534 - 0.855	0.001

Plan Type			

Comprehensive (vs. HMO)	0.917	0.655 - 1.284	0.615

PPO (vs. HMO)	1.173	0.861 - 1.598	0.312

POS (vs. HMO)	0.655	0.424 - 1.013	0.057

Charlson score = 1 (vs. Charlson score = 0)	1.186	0.977 - 1.441	0.085

Charlson score > 1 (vs. Charlson score = 0)	1.146	0.906 - 1.450	0.256

MI listed as the primary diagnosis	1.542	1.329 - 1.788	< 0.001

CABG (vs. MM)	2.677	2.230 - 3.214	< 0.001

PCI (vs. MM)	1.304	1.092 - 1.558	0.003

CABG = coronary artery bypass graft; CI = confidence interval; HMO = health maintenance organization; MI = myocardial infarction; MM = medical management; PCI = percutaneous coronary intervention; POS = point of service; PPO = preferred provider organization.

**Table 6:** Results from multivariate regression for mean total 1-year absenteeism costs

Variables	Cost Ratio	95% CI	p-value
Age Groups			

Age 45-54 (vs. ≤44 years)	1.176	1.005 - 1.376	0.04

Age 55+ (vs. ≤44 years)	0.865	0.698 - 1.072	0.18

Gender			

Female	0.789	0.649 - 0.959	0.02

Plan Type			

Comprehensive (vs. HMO)	0.318	0.195 - 0.517	< 0.0001

PPO (vs. HMO)	0.725	0.571 - 0.920	0.008

POS (vs. HMO)	0.509	0.410 - 0.632	< 0.0001

Charlson score = 1 (vs. Charlson score = 0)	1.145	0.947 - 1.384	0.945

Charlson score > 1 (vs. Charlson score = 0)	0.993	0.824 - 1.198	0.164

MI listed as the primary diagnosis	1.047	0.863 - 1.271	0.639

CABG (vs. MM)	1.415	1.083 - 1.848	0.011

PCI (vs. MM)	0.965	0.837 - 1.113	0.624

CABG = coronary artery bypass graft; CI = confidence interval; HMO = health maintenance organization; MI = myocardial infarction; MM = medical management; PCI = percutaneous coronary intervention; POS = point of service; PPO = preferred provider organization.

## Discussion

This study contributes to the existing medical literature by utilizing more recent data than previously reported ACS cost studies [4-6] to examine both the medical and productivity costs associated with patients treated with MM, PCI or CABG during their ACS index events. This study demonstrated the substantial economic burden associated with ACS in terms of both medical costs and productivity costs, and the costs differed according to treatment strategy (MM, PCI, or CABG) employed during the index event.

The total 1-year medical costs for patients treated conservatively by MM were $34,087, significantly lower compared to patients treated interventionally by PCI ($52,673) or CABG ($86,914). When controlling for patient characteristics, a similar trend was found: the total adjusted 1-year medical costs were $39,017 for MM patients, $58,313 for PCI patients, and $91,977 for CABG patients. All three treatment strategies had higher total 1-year medical costs compared to previous studies resulting from earlier data that reported estimates of $22,528 to $32,345 [4-6]. The higher 1-year medical cost estimates observed in this study compared to the these earlier studies may be due to the general trend of increasing healthcare costs, as well as the use of newer data reflecting changes in treatment strategies along with advances in cardiovascular technology and updated ACC/AHA guidelines. Mortality and outcomes of ACS patients have improved over the last 2 decades due to innovations in pharmacological and interventional treatments in patients, as well as quality improvement initiatives "to move from dealing with individual clinical errors to helping providers to improve the mainstream of care," while focusing on targeted aspects of care including time to reperfusion for STEMI patients and the use of evidence-based therapies during hospitalization, discharge, and postdischarge care [16-18]. Based on a risk stratification approach, the use of PCI or CABG is recommended for high-risk patients presenting with UA/NSTEMI, with a more conservative strategy of MM for low-risk UA/NSTEMI patients [2,7]. Patients that present with STEMI are recommended to receive reperfusion therapy with primary PCI within 90 minutes of initial medical contact [2,8]. While the rates of CABG have remained relatively stable over the years, the rates of PCI have increased, with an estimated 1,313,000 inpatient PCI procedures performed in 2006 [1,2,19].

These results show that PCI and CABG patients had higher total medical costs when compared to MM patients. For both interventional treatments strategies, the majority of the costs occurred during the index event: 59.6% for PCI patients and 73.5% for CABG patients, compared to 26.1% for MM patients. This finding is consistent with earlier studies showing the majority of medical costs resulting from the index event [4-6]. During the 1-year follow-up period, the medical costs for MM patients ($25,182), PCI patients ($21,294), and CABG patients ($23,005) were similar. During the 1-year follow-up period, rehospitalizations accounted for the majority of the medical costs: 45.3% (MM group), 41.2% (PCI group), and 43.5% (CABG). The overall pharmacy costs during the 1-year follow-up period contributed the lowest percentages of the total costs for all three treatment strategies.

To our knowledge, this is the first study examining productivity costs associated with ACS, and the results show the substantial impact on productivity costs. In this study, the productivity costs include short-term disability and absenteeism costs, which are true costs to employers-as employers have to either spend extra resources, such as overstaffing or hiring replacement, to compensate for work normally performed by absent employees or to experience the productivity loss with fewer goods and services provided, lower revenues, and lower profits. In both the short-term disability and absenteeism cohorts, CABG patients had the highest total productivity costs compared to both MM patients and PCI patients. For short-term disability costs, the CABG group had the highest costs within the first 30 days, but had the lowest costs after the first 30 days compared to the MM and PCI groups. In the absenteeism cohort, there was no difference in costs between the three treatment groups during the first 30 days. However, after the first 30 days, CABG patients had significantly higher costs. For all three treatment groups, the majority of the short-term disability costs occurred within the first 30 days of the index event, and the majority of the absenteeism costs occurred after 30 days from the index event.

The treatment of ACS includes an inpatient diagnosis and treatment strategy during the index event, followed by long-term treatment of the underlying coronary heart disease condition. Therefore, it is important to understand the costs associated with different treatment strategies, not only during the index event, but also over time. This study was unique in dividing the costs according to the treatment strategy, MM, PCI, or CABG, employed during the initial ACS hospitalization. The results showed that for PCI and CABG patients, the majority of the costs were incurred during the index event. Costs across all three groups were similar during the 1-year follow-up period. A better understanding of the costs associated with different treatment strategies for ACS patients will help health care practitioners and policy makers in their decision-making process.

Certain limitations must be considered when interpreting these results. Retrospective data analyses were performed; therefore, miscoding of medical claims and missing data, which could not be verified through medical chart reviews, are both possible. In addition, as patients in the study were required to have a minimum 12 months of continuous insurance eligibility after the index date, the results applied only to patients who survived and who did not switch or lose their jobs at least 1 year following their initial hospitalization for ACS. Using an alternative cohort definition, including those patients who lost follow-up during the 12 months after their index events, might have opposite effects on the cost estimation. First, incorporating those who died over the course of 1 year might increase the healthcare costs estimation due to higher end-of-life healthcare. On the other hand, incorporating patients, who experienced job changes or job loss during the one year period after their ACS index events, might lead to lower cost estimation due to loss of insurance eligibility. Mortality status is unknown in the database, unless a patient died in the hospital and the loss of follow-up reason is unclear due to death, job changes, or job loss, etc. As a result, it is unclear how this would impact the cost estimation; therefore, a clear defined cohort was chosen for the study to understand the 1-year medical and productivity costs of ACS to employers.

The productivity cost analyses were limited to short-term disability and absenteeism, and did not include presenteeism (productivity loss due to intermittent reduced output, while at work) or long-term disability. The results from this study may not be generalizable to populations other than working-age populations with employer-sponsored private health insurance plans, such as retired patients or Medicaid beneficiaries.

## Conclusions

In conclusion, this study examined the total 1-year medical and productivity costs associated with short-term disability and absenteeism according to ACS index event treatment strategies, MM, PCI, or CABG. Both total 1-year medical and productivity costs were substantial for working-age individuals with ACS, and these costs differed according to the type of treatment strategy with CABG patients having the highest 1-year medical and productivity costs compared to PCI and MM patients. Treatment options that reduce resource utilization and improve productivity may potentially lower the costs of care for ACS.

MarketScan is a registered trademark of Thomson Reuters (Healthcare) Inc.

## Competing interests

ZZ is employed by Eli Lilly and Company and is a stockholder.

MRW is employed by i3 Innovus.

This project was funded by Daiichi Sankyo Company, Limited and Eli Lilly and Company. The sponsors had no role in the design, methods, data collection, analyses, or preparation of the manuscript. The interpretation and reporting of these data are the sole responsibility of the authors.

## Authors' contributions

ZZ conceived and designed the study and performed the data analyses. MRW acquired the data and contributed to the study conception, design, and data interpretation. Both authors participated in writing the manuscript and have reviewed and approved the final version.

## Appendix

Insurance plan type description:

A variety of insurance plan types was available in the dataset used in this analysis, including fee-for-service (FFS), fully capitated, and partially capitated health plans, including preferred provider organizations (PPO), point of service (POS) plans, comprehensive plans, and health maintenance organizations (HMO). Specifically, in comprehensive plans there is no incentive for the patient to use a particular list of providers. Coverage is handled by one or two policies, with a deductible and coinsurance. Patients who have an HMO plan must choose from a particular list of providers for all non-emergency care. Referral from the patient care provider (PCP) is required for treatment by specialists. All services are paid for by the plan on a capitated basis. In POS plans, patients are offered financial incentives, through a lower copay or deductible, to use a particular list of providers. Referral from the PCP is required for treatment by specialists. Either no or some services are capitated and patients may seek treatment outside the network; however, this option usually involves a severe financial penalty. Patients who have a PPO plan have financial incentives, often through a lower copay or deductible, to use a particular list of providers. No PCP is required, nor are referrals necessary. No services are capitated. Patients may seek treatment outside the network, but this option is usually at a higher cost to the patient. The financial incentives may be offered only through discounted rates in-network.

## Pre-publication history

The pre-publication history for this paper can be accessed here:

http://www.biomedcentral.com/1472-6963/11/35/prepub
